# The “teapot in a city”: A paradigm shift in urban climate modeling

**DOI:** 10.1126/sciadv.abp8934

**Published:** 2022-07-06

**Authors:** Najda Villefranque, Frédéric Hourdin, Louis d’Alençon, Stéphane Blanco, Olivier Boucher, Cyril Caliot, Christophe Coustet, Jérémi Dauchet, Mouna El Hafi, Vincent Eymet, Olivier Farges, Vincent Forest, Richard Fournier, Jacques Gautrais, Valéry Masson, Benjamin Piaud, Robert Schoetter

**Affiliations:** 1 LMD/IPSL/SU, CNRS, Paris 75005, France.; 2 Laplace, INP/Université de Toulouse/CNRS, Toulouse, France.; 3 CNRM, Université de Toulouse, Météo-France, CNRS, Toulouse, France.; 4 LMAP, CNRS, UPPA, E25, Anglet, France.; 5 Méso-Star, Longages, France.; 6 Institut Pascal, Université Clermont Auvergne, Clermont Auvergne INP, CNRS, Clermont-Ferrand, France.; 7 Centre RAPSODEE, Université de Toulouse, Mines Albi, UMR CNRS 5302, Campus Jarlard, Albi, France.; 8 LEMTA, Université de Lorraine, CNRS, Nancy, France.; 9 CRCA, CBI, Université de Toulouse, CNRS, Toulouse, France.

## Abstract

Urban areas are a high-stake target of climate change mitigation and adaptation measures. To understand, predict, and improve the energy performance of cities, the scientific community develops numerical models that describe how they interact with the atmosphere through heat and moisture exchanges at all scales. In this review, we present recent advances that are at the origin of last decade’s revolution in computer graphics, and recent breakthroughs in statistical physics that extend well-established path-integral formulations to nonlinear coupled models. We argue that this rare conjunction of scientific advances in mathematics, physics, computer, and engineering sciences opens promising avenues for urban climate modeling and illustrate this with coupled heat transfer simulations in complex urban geometries under complex atmospheric conditions. We highlight the potential of these approaches beyond urban climate modeling for the necessary appropriation of the issues at the heart of the energy transition by societies.

## Teaser

Statistical physics and computer graphics open new ways for thinking through energy transfers in cities under climate change.

## INTRODUCTION

In the face of global warming, scientists are urged to provide climate information to support mitigation and adaptation policies. To address this challenge, new fields of research that aim at filling the gap between climate change projections and societal needs have emerged. Progress is slow because of the complexity of the systems that need to be analyzed to provide relevant climate information to end users. The questions are multidisciplinary; hence, the expertise of a wide range of communities from climate to human sciences must be involved. The models that are needed to predict the effects of climate change must account for a wide variety of processes characterized by large ranges of spatial and temporal scales. They must be able to ingest large amounts of data from local constraints to climatic records of time-varying meteorological conditions. Uncertainties related to each component must be quantified and propagated through the various model layers. Cities are a high-stake target of adaptation policies and an archetype of such complex systems.

The prime effect of urbanization on the local climate, investigated since the 1980s, is known as the urban heat island (UHI) effect: Cities are almost always warmer than their environment (*1*, *2*). The resulting heat stress, intensified by global warming, leads to health impairment, increased mortality (*3*, *4*), and/or an increase in energy consumption for air conditioning, which positively feedbacks on the UHI (*5*, *6*). As more than half of the world’s population now lives in urban areas (*7*), it has become crucial to adapt cities and design new ones in a way that both improves thermal comfort and reduces energy consumption (*8*, *9*). Climate change mitigation and adaptation measures range from home improvement and renovation by owners, climate-proof building design by architects (*10*), use of new materials and urban cooling technologies (*11*), introduction of urban vegetation (*12*), and exploitation of the surrounding landscape potential (*13*) by urban planners. Identifying and developing urban cool islands has become a priority in some cities. To fulfill this objective, international organizations, such as the World Meteorological Organization, advocate for the development of climate services through which climate scientists are expected to deliver “high-quality, science-based climate information tailored to city requirements to improve urban resiliency and to support the sustainable development of the cities in the world” (*9*).

A classical approach to model climate-related impacts for urban climate services is to rely on either statistical models or urbanized atmospheric models. Accounting for the detailed city geometry, the heterogeneity of urban materials, and the variety of physical processes occurring over a wide range of scales is, however, extremely challenging (*14*). Numerous complementary approaches exist and range from large-scale physical models that account for climate change but markedly simplify the urban geometry (*15*–*21*), to building-resolving models that account for the detailed features of the city but are limited to either small-domain simulations conducted over short time periods (*22*–*28*), or to current climate conditions for statistical models trained on observational datasets (*29*). A brief review of these approaches can be found in Table 1.

**Table 1.** Strengths and limitations of existing urban climate models.

**Physical atmospheric models with parameterized urban canopies**
Transient (e.g., from 1970 to 2100) global or regional climate simulations can be made at 10- to 100-km resolution using atmospheric models (*15*). Atmospheric models at hectometric to kilometric resolution can provide simulations for a few days and up to 1 year (*74*, *75*). In both cases, the cities cannot be represented explicitly. Rather, urban canopy models like the Building Effect Parametrization (BEP) (*16*) or the Town Energy Balance (TEB) (*17*) [including a building energy model (*18*–*20*)] are used to estimate the effect of subgrid radiative and heat transfer on the air temperature, winds, and water balance. The geometry of the city is greatly simplified, usually using the “urban canyon” approximation [an infinite street with two facing walls (*21*)]. These models provide useful information such as the impact of the urban heat island on the building energy consumption, sometimes in an operational service such as in Beijing (*76*). However, they cannot provide information at the scale of a flat or a building, nor do they help to assess the impact of small-scale adaptation measures.
**Physical building-resolving models with parameterized** **environment**
Higher-resolution building-resolving micrometeorological models can represent the detailed urban geometry, but simulations are limited to a neighborhood (typically 500 m by 500 m) and simulations up to a few days (*22*, *23*). Building energy models like EnergyPlus (*24*) simulate the energy budget of an individual building, accounting for a high level of detail [e.g., room allocation, building occupant behavior (*25*), and types of shading elements]. They rely on other models such as CITYSIM (*26*) or SOLENE (*27*, *28*) to model the environmental effects, like the shading of adjacent buildings. In this approach, investigation of the impact of climate change is severely limited by the difficulty to handle meteorological forcings.
**Statistical models**
They are usually trained on local observations and limited to the site and conditions under which observations are available. They sometimes use statistical laws calibrated on various sites to provide estimations of quantities on other neighborhoods [e.g., the Urban Multi-scale Environmental Predictor (UMEP) (*29*)]. They are computationally efficient but assume constant statistical relationships between historical and future climate. They are limited to the resolution of the observational data, although the impacts of processes occurring at all scales are inherently integrated into the measurements that constitute the training dataset.

**Fig. 1.**
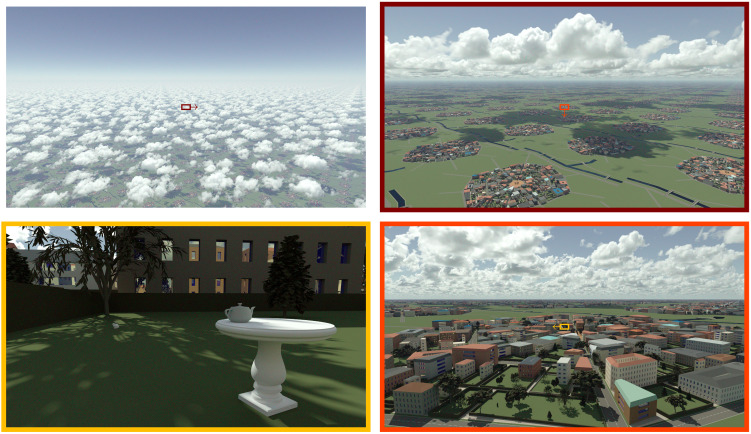
The teapot in a city under cumulus clouds, in reference to the “teapot in the stadium” problem. The four pictures are sampled from an animated movie (www.lmd.jussieu.fr/~nvillefranque/pages/teapot_city) we produced using the htrdr model (*62*) (www.meso-star.com/projects/htrdr/htrdr.html) that solves radiative transfer in the atmosphere and in cities. Each image features a different cloud field, camera, and sun positions. Periodic conditions were used for the city geometry and the cloud fields to demonstrate insensitivity to the scene dimension. Cities and cloud fields of larger extent can be rendered with open boundary conditions as easily, provided that the data are available. The urban geometry was generated using a procedural generator (https://gitlab.com/meso-star/city_generator) based on sampling distributions that represent the building characteristics (height, spacing, ...) and various tree geometries. The spectrally varying radiative properties of the materials were taken from the Spectral Library of Impervious Urban Materials (SLUM) database (*77*). The cloudy atmosphere was simulated using the Meso-NH large-eddy simulation (LES) model (*75*, *78*) and represents a typical fair-weather cumulus field evolving over a flat ground (*79*) at 8-m resolution on a 15 km × 15 km **×** 4 km domain with horizontally periodic boundary conditions with 3D fields output every 15 s between 11:30 and 13:00 local solar time (LST).

In this study, we present a new paradigm for multiscale modeling of coupled radiative and heat transfer in complex urban geometry under changing climate. It relies on probabilistic models solved by Monte Carlo methods and builds on recent advances in computer graphics. The “teapot in a stadium” problem (*30*), namely, the difficulty to render small-scale details (the teapot) within a very large scene (a stadium), has been solved (*31*). The computing time associated with path-tracing in three-dimensional (3D) scenes is now close to insensitive to the scene complexity. As Monte Carlo methods used to solve the radiative transfer equation are independent from the description of the geometric data, increasing the computation accuracy can be achieved by improving the physical model or improving the data in completely independent developments. In the world of 3D animation for film production, this property has freed up the artists who do no longer have to compromise on the complexity of their scenes to comply with the limitations of rendering algorithms. Similarly, computer scientists have been able to include more complex physics in their algorithms, producing ever more realistic images by taking into account every detail of the virtual scene in a physically consistent manner. We illustrate this in Fig. 1 with four images sampled from the animated movie of a “teapot in a city under cumulus clouds,” available at www.lmd.jussieu.fr/~nvillefranque/pages/teapot_city. It is based entirely on physical principles, both for simulating clouds and for light propagation.

We envision that the exact same framework of formulating physical processes as path integrals and integrating them numerically with Monte Carlo path-tracing methods could lead to a similar revolution in urban modeling. Here, we review recently published results that, put together, allow for computations that were previously unthinkable. Before reviewing these breakthroughs and reflecting on the perspectives that are opening up for urban climate services, we present the foundations of these methods using a very simple example of computing the energetics of a 2D building. In doing so, we hope to introduce the readers to the key concepts of the framework, from the most technical aspects to their most profound implications; we also illustrate the benefits of the framework for the analysis and understanding of complex systems.

## A SIMPLE EXAMPLE OF PATH-INTEGRAL FORMULATION

**Fig. 2.**
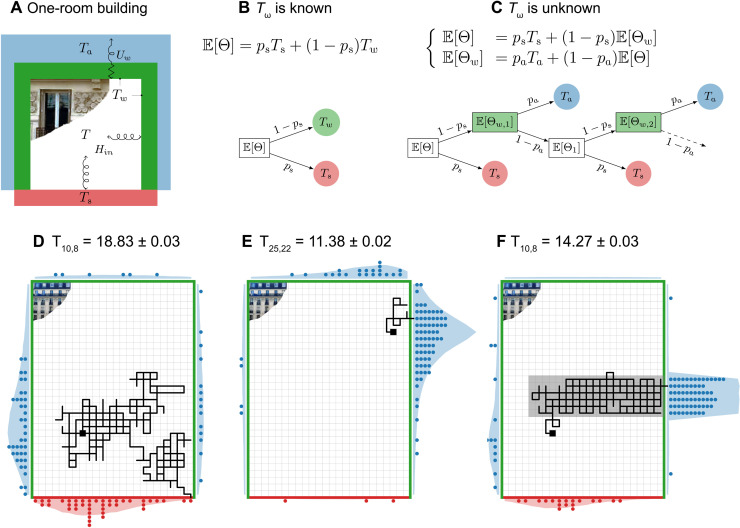
Idealized heat transfer in 2D buildings. (**A** to **C**) Single room building. (**D** to **F**) N x M rooms building. (A) *T* is the temperature of the room’s perfectly mixed air, *T*_s_ is the temperature of the ground floor, *T*_w_ is the temperature of the three other walls, and *T*_a_ is the temperature of the environmental perfectly mixed air. Heat exchange between the inside air and the interior walls is driven by convection, with convective thermal conductance (CTC) *H*_in_. Heat exchange between the interior walls and the outside air is driven by conduction in the wall and convection outside, of global thermal conductance *U*_w_. (B) *T* is the average of *T*_w_ and *T*_s_, which is also the expectation of Θ whose outcomes are *T*_s_ with probability *p*_s_ and *T*_w_ with probability 1 − *p*_s_. (C) *T*_w_ is itself the expectation of Θ_w_. One realization of Θ is sampled by first sampling Θ_w,n_ and then Θ_n_ successively until an outcome (*T*_s_ or *T*_a_) is found. (Θ_w,n_)_*n* = 1, …*N*_ and (Θ_n_)_*n* = 1, …*N*_ are collections of independent and identically distributed random variables that have the same probability law as Θ_w_ and Θ, respectively. (D to F) *T*_*i*,*j*_ is the temperature in room (*i*, *j*) (black square). The exterior walls have the same properties as in (A), *T*_a_ = 10^°^C, *T*_s_ = 30^°^C. The interior walls all have the same CTCs except in the gray zone of (F) where they are a hundred times larger, which is symptomatic of a thermal bridge. The first sampled path (black line), the end locations of the first 100 sampled paths (blue and red points), and the distribution of the end locations of the 100,000 sampled paths (blue and red shadings) are shown for each simulation.

The framework we present here is built on two fundamental ideas: the formulation of deterministic physical models as integrals over path spaces (*32*, *33*) and double randomization (*34*). Let us illustrate them for a simple 2D model of the steady-state temperature *T* of perfectly mixed air inside a square room framed by three segment walls (temperature *T*_w_) and ground floor (temperature *T*_s_), surrounded by air at temperature *T*_a_ (see schematic in Fig. 2A). Per analogy with an electrical network, *T* can be written as the average of the ground and walls’ temperature weighted by the wall convective thermal conductances (convective coefficients times length of the wall). If the room is a square and the convective coefficient is constant, then *T* = *p*_s_*T*_s_ + (1 − *p*_s_)*T*_w_ with *p*_s_ = 1/4.

Let us shed a probabilistic light on this deterministic problem and interpret *T* as the expectation of a random variable Θ following a Bernoulli’s law of parameter *p*_s_, with outcomes *T*_s_ and *T*_w_ (see Fig. 2B). An unbiased estimate of *T* can then be produced by averaging a large number of realizations of Θ. More generally, whenever it is possible to formulate a quantity as an expectation of a (function of) discrete or continuous random variable(s), then this quantity can be estimated using Monte Carlo methods. This is the first fundamental idea of the framework.

Now, let the value of *T*_w_ be unknown. Defining a global thermal wall conductance to account for conduction in the wall and convection oustide, and considering that heat fluxes are continuous at the inner wall surface, *T*_w_ can again be written as the expectation of a random variable following another Bernoulli’s law of parameter *p*_a_, with possible outcomes *T*_a_ and *T* (see Fig. 2C).

Combining the expressions for T=E[Θ] and $T_{w}=E[\Theta_{w}]$ yields a recursive expression. Most of the time, there is no closed-form equivalent for the recursive expressions that come from the probabilistic formulation of a deterministic problem; therefore, the “global” law that directly gives the probability of the final outcomes (*T*_a_ or *T*_s_) cannot be sampled. This is where the concept of double randomization is needed. It consists of sampling the “local” probability laws (of Θ and Θ_w_) successively until finding an outcome (*T*_a_ or *T*_s_) for the random sequence or path. This is illustrated in Fig. 2C. The justification for double randomization is mathematically trivial (it comes from the law of total expectation), albeit conceptually subtle. This second fundamental idea explains why Monte Carlo methods are insensitive to the problem’s dimension: Each step of the sampling procedure is entirely oblivious to the rest of the model.

Now, let the building consist of many rooms (*i*, *j*) at temperature $T_{i,j}=E[\Theta_{i,j}]$ (see Fig. 2, D to F) with known thermal conductances and boundary conditions (*T*_s_ and *T*_a_). Implementing the same strategy as before to compute *T*_*i*,*j*_ yields a Monte Carlo algorithm that consists of successively sampling neighboring rooms, starting at (*i*, *j*), until finding an outcome (a boundary condition). *T*_*i*,*j*_ is then estimated as the mean outcome. One realization of Θ_*i*,*j*_ can be represented as a path throughout the building from room (*i*, *j*) to the location of the outcome, as in Fig. 2 (D to F). Randomly constructing these paths step by step using double randomization ensures that the two possible outcomes are sampled in the correct proportions. Figure 2 (D to F) displays the distribution of the path outcomes; more paths end in the neighborhood of the (*i*, *j*) room than at opposite walls, except in Fig. 2F where the paths show that most of the heat is lost through the poorly insulated part of the building.

The building heat loss is proportional to the difference between the outside air and the average temperature of the boundary rooms of the building. Estimating this average temperature instead of the temperature of one particular room can be done using the same algorithm, except that instead of fixing (*i*, *j*) beforehand, a starting room is randomly sampled at the beginning of each path. As “probe” type computations do not rely on solving the entire field, the quantity is obtained at a point or on average for approximately the same computing time.

## BREAKTHROUGHS IN MONTE CARLO METHODS

A major feature of Monte Carlo methods is their insensitivity to the dimension of the problem. In the 2D building of Fig. 2, computing the heat loss of the *N* × *M* room building at 1% precision takes roughly the same computing time as computing the heat loss of a building twice as high, made of twice as many storeys. This feature has been central in the use of these methods throughout many scientific fields ever since Nicholas Metropolis and Stanislaw Ulam coined the name “Monte Carlo method” in their famous 1949 article (*35*). It is the same feature that makes Monte Carlo methods so powerful to solve recursive equations such as the Fredholm equations of the second kind, and extensively used in particle transport from neutronics to rarefied gas to radiative transfer (*36*). After use of kernel iterative method, the Fredholm equation admits Von Neumann series representation making the integration problem of infinite dimension (*37*, *38*), but double randomization simply translates it into successive sampling collision events one after the other (much as successive neighboring rooms are sampled in the example of the 2D building) until an outcome is found. A major step was achieved when Monte Carlo methods were extended to problems that were not initially formulated into the framework of statistical physics. This led to important advances in the field of applied mathematics where Monte Carlo methods are now routinely used for large matrix inversion, and in physics when Richard Feynman and Marc Kac formulated the general solution of the differential equations that model advecto-reacto-diffusive processes as the expectation of a Wiener process (*32*, *33*, *39*–*41*). This opened previously unexplored fields of application with, for instance, Monte Carlo simulations of Brownian motions to solve 3D transient diffusion (*42*–*45*).

However, the insensitivity to the dimension was lost when problems included either (i) 3D geometries characterized by wide ranges of scales, (ii) coupled models of different natures, or (iii) nonlinearities. The three following breakthroughs could overcome these limitations.

Breakthrough (i) was achieved by the computer graphics community who has invested and revolutionized the field of Monte Carlo physically based rendering (*46*–*49*). To increase the realism of animated movies, they increased the geometric details of the rendered virtual scenes, thus increasing the number of facets to be tested for intersection when tracing paths. That led them to conceive hierarchical structures to organize the data in memory so that the cost of path-tracing became independent of the number of facets describing the scene (*31*, *50*–*52*). Until recently, however, ray tracing procedures were inefficient in highly heterogeneous media such as clouds, due to the nonlinearity of Beer’s exponential law. A first step toward efficiency was to make ray tracing independent from the description of the medium with null-collision algorithms (*53*, *54*). This opened new possibilities that began to be investigated in physics (*55*–*58*) and computer graphics (*59*–*61*). From there, the hierarchical structures were extended to handle complex volumetric data (*62*), thereby making the cost of numerical computations in cloudy atmosphere insensitive to the details of the cloud description. This first breakthrough is illustrated in Fig. 1. In each image, all the details of the clouds and the city, including the teapot, are taken into account, even when they are not perceivable to the eye.

**Fig. 3.**
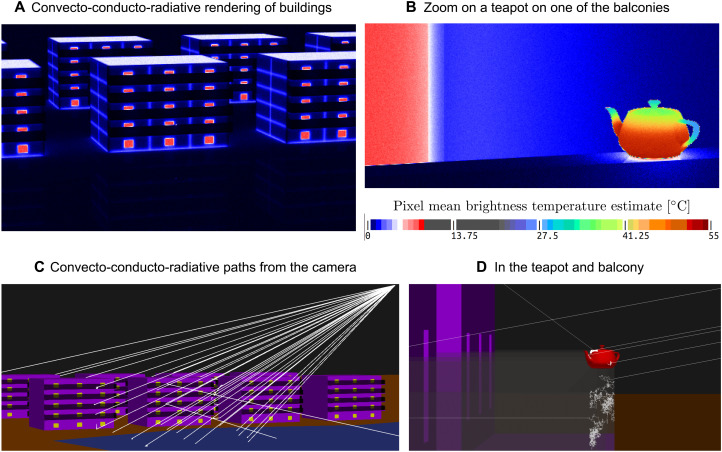
Physical infrared rendering of 3D buildings near a lake, in steady state, at night. The brightness temperature equivalent to the radiation emitted by the buildings, ground, and atmosphere and received at the virtual camera is computed in each pixel by solving detailed heat transfers in the scene, using the Stardis software (http://meso-star.com/projects/stardis/stardis.html). Paths start at the camera; conduction is simulated using δ-sphere walks inside the solids; radiative exchanges are sampled between surfaces. Paths stop upon reaching a boundary condition: the temperature of the atmosphere (0°C), and rooms (20°C) by convection or the brightness temperature of the atmosphere (0°C) by radiation. They can also stop in the teapot that contains water at an imposed temperature of 60°C. (**A** and **B**) Results of convective-conductive-radiative Monte Carlo simulations for two views: (A) a few buildings and (B) a zoom on the teapot. Note that in (A), the teapot is already on the first floor balcony of the middle building; it increases the mean brightness temperature of one of the pixels inside the red frame. (**C** and **D**) 3D visualization of the scene and of a few paths sampled during the simulations. The scene consists of 33,958 facets (10,234 facets to describe the teapot and 23,724 for the buildings). Each image consists of 480× 280 independent Monte Carlo estimates (one per pixel, 512 paths each).

Breakthrough (ii) was to understand that the double randomization concept enables the coupling of models with no theoretical limit to the number, nature, or scale of the represented processes (*63*). As an illustration, Fig. 3 displays a virtual infrared image of buildings at night time, which was rendered by tracing paths from a camera solving a coupled conductive-convective-radiative equation. The continuity of the boundary fluxes is reformulated as a probability to switch from one transfer mode to the other, much as in the example of the 2D building. In this example, the temperature of the rooms in the building is 20^∘^ and the outside air temperature is 0^∘^. Thermal losses through windows, roofs, and floors are captured by the infrared virtual camera (Fig. 3A) by explicitly simulating heat transfer processes in the detailed geometry. A few brighter pixels at the center of the image are due to the presence of a hot teapot set on the balcony, as revealed by the zoomed image of Fig. 3B. The teapot is half filled with hot liquid, creating a temperature gradient on the teapot surface, with warm bottom and colder lid, pout, and handle. These fine-scale details were captured by the paths simulated inside the teapot geometry, with some of the paths also exploring parts of the geometry that are not directly visible to the camera, capturing the larger-scale transfers shown in Fig. 3A. The teapot is an iconic object from the computer graphics community; it has no climatic relevance but here serves to demonstrate that large ranges of scales can be seamlessly integrated. The thermal loss integrated over the entire city can be computed in the same framework; because the temperature field never needs to be estimated, the computational expense will be approximately the computing time associated with one pixel of the images. Other coupled models have been solved on the basis of the same idea, such as the radiative transfer equation coupled with a spectroscopy model, directly integrating the spectral lines, thereby avoiding the heavy precomputation of absorption coefficient spectra (*56*). It was also used in process engineering to solve a cascade of embedded models from radiative transfer to electromagnetism to thermokinetic coupling to spectroscopy, to estimate the biomass production of a photoreactor system at the industrial scale (*64*). The paths are no longer restricted to the 3D space but “travel” through models of different natures.

Although “direct simulation Monte Carlo” methods (*65*) have for long addressed nonlinear physics by simultaneously tracing large numbers of paths so that the tracked particles could interact with each other, they are fundamentally sensitive to the model or domain dimensions: Increasing the accuracy of the estimates implies increasing the particle density everywhere in the spatial domain and the other domains of integration of the problem. Recent advances have paved the way for nonlinear Monte Carlo calculations that preserve the insensitivity property [breakthrough (iii)]. First, and although atmospheric radiative transfer is fundamentally linear, the null-collision approach mentioned above can be seen as a way to bypass Beer’s nonlinearity (*66*). It has been shown that other types of nonlinearities could be treated using ramified paths (*67*, *68*). The algorithms are more complex, but the paths can still be sampled independently, thus preserving all the benefits of probe Monte Carlo approaches. Further investigations have shown that iterative methods could be used to limit the recursivity level of the path ramifications, enhancing the practicability of the method (*69*).

## IMPLICATIONS FOR URBAN CLIMATE SERVICES

**Fig. 4.**
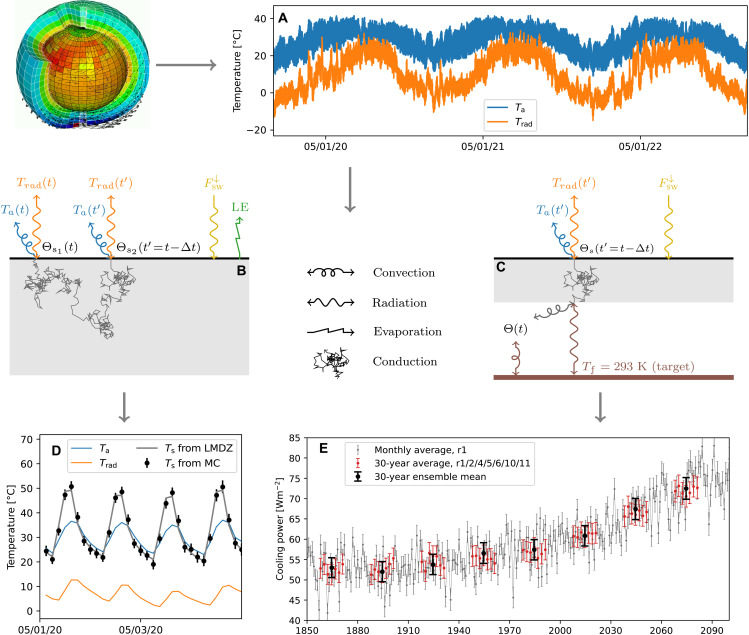
Time-varying meteorological conditions are used as inputs and parameters in path-integral heat transfer models. (**A**) Air temperature at 2 m above the surface (*T*_a_) and the atmospheric brightness temperature (*T*_rad_) issued from a climate change simulation performed with the IPSL-CM6A-LR global model (*80*), available at a 3-hour frequency over 250 years. The variables retrieved from the climate archive are the following: *T*_a_, the downwelling longwave ($F_{\text{LW}}^{↓}$, used to compute *T*_rad_) and shortwave ($F_{\text{SW}}^{↓}$) radiative fluxes at the surface, the sensible (*H*) and latent (LE) turbulent heat fluxes, and the surface temperature *T*_s_. *H* and *T*_s_ are used to compute a convective exchange coefficient *h* = *H*/(*T*_s_ − *T*_a_). LE and $F_{\text{SW}}^{↓}$ are imposed fluxes. The data correspond to a gridpoint in Sahel. (**B** and **C**) Random path representation of the heat transfer models used to estimate (D) the surface temperature of a homogeneous soil of thermal inertia 1500 J m^−2^ s^−1/2^ K and (E) the air-conditioning power to maintain a simplified room’s floor at 293 K. (**D**) Instantaneous temperatures every 3 hours during 4 days: Monte Carlo estimates of *T_s_* (black dots) and *T*_s_, *T*_a_, and *T*_rad_ from the climate archive (gray, blue, and orange lines). (**E**) May averages of air-conditioning power from 1850 to 2100: every year (gray dots); averaged over 30 years (red dots), each red dot corresponds to a different member of an ensemble simulation (*71*); averaged over 30 years and over the ensemble members (black dots). Dots and error bars in (D) and (E) correspond to Monte Carlo estimates based on 30,000 paths and their associated 99.7% confidence interval.

To inform adaptation strategies aiming at minimizing urban heat stress and energy consumption, the performances of new construction materials, building, and city designs need to be assessed in realistic environmental conditions representative of future climates, at various spatiotemporal scales, with unconstrained amounts of geometric details. The properties of Monte Carlo probe computations open an avenue for these computations by offering the possibility to sample weather conditions in addition to the other dimensions of the problem (*70*). Figure 4 illustrates in a simple geometry how the approach allows to integrate the large temporal scale factors from the meteorological process scale to the climate scale in a relevant multiphysics calculation. Outputs of an ensemble of global climate simulations of 250 years each are used, available at a frequency of 3 hours (*71*).

In the first example, the estimated quantity is the temperature at the surface of a homogeneous soil at a given time (Fig. 4, B and D). Paths start at the soil’s surface and travel through the system and backward in time. Each time the path encounters the surface, solar and evaporative heat fluxes are added to the Monte Carlo weight and the flux continuity equation gives the probability that the path goes into the atmosphere by convection or infrared thermal radiation (the path ends with outcome *T*_a_ or *T*_rad_, respectively) or penetrates the ground by conduction.

Transient conduction is simulated using diffusive random walks. At each step, the duration associated with the step length is sampled from an exponential law parameterized by the material inertia, as per the Green first-passage times distribution function. The walk goes on until the initial condition (at year 1850 here) or the surface is reached. Encounters with the surface will therefore happen at different times depending on the random duration of the walk. It is only at these times that the meteorological data need to be accessed. The time dimension is thus sampled based on the physical properties of the system: The longest paths will go back farther in time if the thermal inertia of the ground is larger. Moreover, the influence of the meteorology onto the surface temperature is sampled according to the detailed meteorological processes. For instance, the probability for a path to end up in the air by convection is generally smaller during nighttime than during daytime because of a smaller value of the convective exchange coefficient.

The second example (Fig. 4, C and E) is the computation of the air-conditioning power needed to maintain a room’s floor temperature to a set point of 20°C. In the upper part of the geometry (atmosphere + roof), the model is the same as in the previous example except that latent heat fluxes are neglected. In the lower part, the temperature at the bottom of the slab roof (i.e., the ceiling) is coupled to that of the (perfectly mixed) room’s air by convection and to that of the floor by radiation. The air-conditioning power is calculated as the net heat flux between the floor and the system.

Using double randomization, a single Monte Carlo simulation is used to estimate the power not at a particular time but on average over a given period. This is achieved by sampling a different starting time at the beginning of each path. Because this additional sampling does not increase the sample variance, the same number of paths, and hence the same computing time, is needed to reach a 1% accuracy as for a single-time estimate. Sampling the members of an ensemble of climate simulations also gives an estimate of the power averaged over the ensemble of simulated meteorological stories, again at the same cost as for a single-member single-time computation.

This very preliminary computation obviously suffers from several limitations. The building geometry is oversimplified compared to Fig. 3, as is the treatment of atmospheric radiation compared to Fig. 1. First, the codes that have been used to produce these images (stardis and htrdr) still need to be coupled together and interfaced with the climate data. Important work remains to produce geometric data at the required format in a way that is flexible enough to allow simple user modifications. Second, the temperature of the near-surface air (used to compute turbulent or convective fluxes) and the downward thermal radiative fluxes are unaffected by the temperature of the building, which prevents the representation of the UHI effect. Third, in contrast to urban canopy models or obstacle resolving models that are often based on the resolution of fluid dynamics but struggle to integrate a full description of the thermal transfers in the buildings, the Monte Carlo methods easily solve the physical and geometrical complexity of the thermal and radiative transfer in the buildings but struggle to solve the atmospheric flow.

A research program is currently funded by the French National Agency for Research to overcome these limitations. The perturbation of the air temperature above the buildings will indirectly be taken into account by pursuing the paths in the atmosphere through turbulent, convective updrafts or advective motions. The path will end with the air temperature from the model only once the path is outside the city’s footprint, thereby representing the UHI. On the other hand, the Monte Carlo path-tracing algorithm that solves the thermal exchanges in the city will be coupled to an obstacle resolving model of the flow in the city. For this coupling, the temperature has to be estimated at all the building interface with the atmosphere; hence, alternatives to the classical probe computation are studied to accelerate the computation such as the symbolic Monte Carlo methods (*57*). Only relatively short simulations will be produced this way, to serve as a reference to benchmark faster models; this strategy was already proven successful for the development of cloud parameterizations, using explicit large-eddy simulation (LES) as a reference (*72*).

Note that for adaptation issues, these computations should rely on global coupled climate models, the only models able to simulate the thousands of years of meteorological evolution that are required to achieve equilibrium and simulate climate change. The representation of convective and cloud processes in these models has strongly improved in the last decades because of recent advances in parameterizations and model tuning (*73*), although much work remains to reduce the uncertainties associated with the representation of these processes. Because of persisting biases in global models and of their rather coarse resolution, statistical or dynamical downscaling, for instance, using regional climate models, might prove necessary to better account for local constraints or detailed processes such as topography or the radiative effect of geometrically complex clouds. This opens exciting questions that are yet to be investigated.

A recurrent challenge of climate services is that climate records and simulations represent huge amounts of data from which information relevant to the user’s needs has to be extracted, preferably in a comprehensible, flexible way so that end users can be in full responsibility of their work. Here, the idea is to run probe computations taking the full history of the atmospheric column as an input, provided by global or regional climate simulations. No pretreatment of the data is needed: Through path integrals, the physics define a relevant way to aggregate the climate data. Flexibility is ensured by the fact that each quantity to be estimated will be associated with its own path-integral, its own tailored “data mining” procedure. This implies that the data output from climate simulations must be made entirely available, which is in line with the open-science (open-source and open-data) philosophy. It can, of course, raise practical issues, slowing the computations down if data access is not managed efficiently. Packaging software in containers to be run on servers where the data are stored, or downloading a single column of the full history of climate simulations beforehand as was done for the simple illustration of Fig. 4, might be adequate solutions.

An important aspect of our proposition is the empowerment of users. As for 3D animation, this framework is intended to set free the users who could add any details to the building designs and materials without fearing consequences on the numerical cost of their choices, because of the independence between the algorithms and the data description. Moreover, we believe that the paths convey meaningful images that have the potential to enlighten scientists and nonscientists with intuitive understanding of the physical processes at stake and their interactions. The theoretical framework and associated numerical tools provide more than numbers; they provide insights into the questions at the heart of the energy transition.
